# Disparities in Functional Outcome After Intracerebral Hemorrhage Among Asians and Pacific Islanders

**DOI:** 10.3389/fneur.2018.00186

**Published:** 2018-03-27

**Authors:** Kazuma Nakagawa, Sage L. King, Todd B. Seto, Marjorie K. L. M. Mau

**Affiliations:** ^1^The Queen’s Medical Center, Honolulu, HI, United States; ^2^Department of Medicine, John A. Burns School of Medicine, University of Hawaii, Honolulu, HI, United States; ^3^Department of Native Hawaiian Health, John A. Burns School of Medicine, University of Hawaii, Honolulu, HI, United States

**Keywords:** race, functional outcome, intracerebral hemorrhage

## Abstract

**Background:**

Disparities in outcome after intracerebral hemorrhage (ICH) among Asians, Native Hawaiians, and other Pacific Islanders (NHOPI) have been inadequately studied. We sought to assess differences in functional outcome between Asians and NHOPI after ICH.

**Methods:**

A multiracial prospective cohort study of ICH patients was conducted from 2011 to 2016 at a tertiary center in Honolulu, HI, USA to assess racial disparities in outcome after ICH. Favorable outcome was defined as 3-month modified Rankin Scale (mRS) score ≤2. Patients with no available 3-month functional outcome, race other than Asians and NHOPI, and baseline mRS > 0 were excluded. Multivariable analyses using logistic regression were performed to assess the impact of race on favorable outcome after adjusting for the ICH Score, early do-not-resuscitate (DNR) order and dementia/cognitive impairment.

**Results:**

A total of 220 patients (161 Asians, 59 NHOPI) were studied. Overall, 65 (29.5%) achieved favorable outcome at 3 months. NHOPI were younger than Asians (*p* < 0.0001) and had higher prevalence of diabetes (*p* = 0.007), obesity (*p* < 0.0001), and lower prevalence of dementia/cognitive impairment (*p* = 0.02), early DNR order (*p* = 0.0004), and advance directive presence (*p* = 0.0005). NHOPI race was a predictor of favorable outcome in the unadjusted model [odds ratio (OR) 2.47, 95% confidence interval (CI): 1.32–4.62] and after adjusting for the ICH Score (OR 2.30, 95% CI: 1.06–4.97) but not in the final model (OR 2.04, 95% CI: 0.94–4.42). In the final model, the ICH Score was the only independent negative predictor of outcome (OR 0.26, 95% CI: 0.17–0.41 per point).

**Conclusion:**

NHOPI are more likely to achieve favorable functional outcome after ICH compared with Asians even after controlling for ICH severity. However, this association was attenuated by the DNR and dementia/cognitive impairment status.

## Introduction

Intracerebral hemorrhage (ICH) has disproportionately high mortality and morbidity with only 21–31% of patients gaining functional independence at 3 months (1–3). Prior studies that assessed racial/ethnic disparities in ICH have shown that minority groups, particularly African Americans and Hispanics, have a higher incidence of ICH and younger age of presentation than whites (4, 5). Asians are also estimated to have a higher incidence of ICH compared with Caucasians (6). In Hawaii, Native Hawaiians and Other Pacific Islanders (NHOPI), have been shown to have younger age of ICH onset and higher burden of cardiovascular risk factors compared with whites (7, 8).

Although the overall incidence of stroke is greater among minorities, the reported impact of ethnicity on outcomes after stroke has been inconsistent and unclear. Prior studies showed greater initial physical impairments and greater residual disability in African American stroke patients compared with white stroke patients (9, 10). Another study showed that blacks had severer cases and higher prevalence of coma after ICH (11). However, more recent studies have shown comparable or better outcomes after ICH among blacks and Hispanics compared with whites (3, 12). A few studies that specifically assessed Asians showed lower risk-adjusted mortality after ICH and ischemic stroke compared with whites.

Overall, there is still a paucity of data on racial disparities in ICH outcome among Asians and NHOPI. Furthermore, NHOPI have been historically aggregated with Asians into a single racial category in many studies. Since Asians and NHOPI may have different underlying phenotype, lifestyle, socioeconomic status and different level of acculturation to the western civilization, aggregating them into a single racial group may have masked potentially important differences between the two groups (13). Therefore, we sought to compare the functional outcome after ICH between Asians and NHOPI. We hypothesized that Asians, a racial group that has higher life expectancy and lower cardiovascular risk factors than NHOPI, are more likely to have better functional outcome after ICH compared with NHOPI.

## Patients and Methods

A multiracial prospective cohort study of ICH patients (Queen Emma Stroke Study) was conducted from July 2011 to June 2016 at The Queen’s Medical Center (QMC) to assess racial disparities in long-term functional outcome after ICH. Last patient enrollment occurred in June 2015, and last follow up assessment occurred in June 2016. QMC is a 505-bed medical center located in downtown Honolulu, the largest hospital in Hawaii and the tertiary referral center for the Pacific Basin. QMC is a Joint Commission-certified Comprehensive Stroke Center and has the only Neuroscience Intensive Care Unit in the state of Hawaii. Since ICH is a condition that is preferably treated in the Neuroscience Intensive Care Unit, QMC is the primary referral center for acute management of ICH patients from other major islands. This study was approved by the University of Hawaii Institutional Review Board, and written informed consent was obtained from the patient or legally authorized surrogate decision-maker.

### Patients

All ICH patients hospitalized at QMC were prospectively screened for enrollment by the investigators and research staff. Inclusion criteria for the cohort study included: age ≥18 years; non-traumatic ICH with confirmation by computed tomography (CT); resident of Hawaii for greater than 3 months in a household with a telephone. Exclusion criteria were: ICH related to ruptured cerebral aneurysm; ICH related to brain tumor or hemorrhagic conversion of ischemic stroke, and the patients or surrogate decision-makers being non-English speaking. Management of ICH at QMC was in accordance with the most current guidelines (14) at the time of patient enrollment.

### Study Procedures

This was an observational study and no intervention was provided. Patient demographic information (race, ethnicity, marital status, income level, insurance status, etc.) was obtained directly from the patients or their family if the patients were incapacitated. Since mixed racial background is relatively common in Hawaii, race was defined as the single racial/cultural background that the patient most closely associated with and was based on patient self-identification or family’s identification if the patient was incapacitated. Race was ultimately categorized as whites, Asians, NHOPI or other. Clinical diagnosis of preexisting dementia or cognitive impairment had to be made by their respective physicians before the hospitalization through formal clinical evaluation. Initial systolic blood pressure, diastolic blood pressure (DBP), and Glasgow Coma Scale (GCS) score were obtained from the medical record. Obesity was defined as body mass index >30 kg/m^2^ for all racial groups. Regular visit to a primary care physician was defined as a minimum of one routine visit per year over the past year. Prehospital baseline functional status was assessed using the modified Rankin Scale (mRS) and was obtained through direct patient/family interview. All initial head CT scans were reviewed by one of the physician investigators (Kazuma Nakagawa) using a standardized protocol. Hematoma volume was measured using the previously described ABC/2 method (15). Presence of intraventricular hemorrhage (IVH) was recorded, and ICH location was coded as deep (basal ganglia or thalamus), lobar, brainstem, cerebellum, or primary IVH. The ICH Score was calculated based on the age, initial head CT findings, and the initial GCS on arrival. Do-not-resuscitate (DNR) orders were defined as any plan to limit cardiopulmonary resuscitation or mechanical ventilation in the event of a cardiopulmonary arrest. Based on the date/time of the DNR order entry, DNR status was categorized to “early DNR” (within 24 h of presentation) or “any DNR” (DNR orders at any time). Presence of advance directive was confirmed by its physical presence in the paper chart and documentation of its content by the primary provider.

### Outcome Measures

Outcome at 3 months was assessed by telephone or in-person interview using a standardized, simplified mRS questionnaire algorithm (16). Favorable outcome was defined as 3-month mRS score ≤2.

### Statistical Analysis

Data were analyzed using SPSS version 23.0 (SPSS IBM Inc., Chicago, IL, USA). For this study, patients with no available 3-month functional outcome and patients with race other than Asians and NHOPI were excluded. Although we had initially planned to include whites as a reference racial group for the study, whites were ultimately excluded from the analysis since only 37 whites were enrolled in the study, and their inclusion would have weakened the statistical analyses and generalizability. Finally, patients with baseline mRS > 0 were excluded since preexisting disability would have confounded the results. Patient characteristics were summarized using descriptive statistics appropriate to variable type. NHOPI were compared with Asians (reference group) using the chi-square test for categorical data, two-tailed *t*-test for normally distributed, continuous variables, and Mann–Whitney *U* test for non-parametric data (i.e., age, blood pressure, GCS, and hematoma volume). To determine the impact of race on outcome, multivariable analyses using a logistic regression model were performed to assess whether NHOPI race, compared with Asian race, is an independent predictor of 3-month functional outcome. Due to the small sample size, which limited our ability to include numerous variables in the model, we had preselected three potential confounders in the model: ICH Score, early DNR order status and history of dementia/cognitive impairment, based on biological plausibility and prior studies showing association between these factors and functional outcome after ICH (1, 2, 17, 18). For secondary analyses, a similar process was repeated after excluding the in-hospital non-survivors to eliminate the impact of in-hospital mortality as the primary factor driving the potential racial differences in 3-month outcome. Odds ratio (OR) and 95% confidence interval (CI) were calculated from the beta coefficients and their SEs. Levels of *p* < 0.05 were considered statistically significant.

## Results

A total of 323 ICH patients were enrolled in the cohort study between July 2011 and June 2015. Among them, 32 patients who did not have 3-month outcome data were excluded from the analyses. Of the remaining 291 patients, 37 patients with white race and 9 with other race were excluded due to small population size. Furthermore, 25 patients with baseline mRS > 0 were excluded. Table 1 provides the demographic and clinical information of 220 patients [Asians 73.2% (*n* = 161), NHOPI 26.8% (*n* = 59)] who were included in the final analyses. Overall, NHOPI had younger age (12.1 years younger), lower socioeconomic status, lower rate of regular visit to their primary care physician, higher prevalence of diabetes and obesity, higher initial DBP and lower prevalence of dementia/cognitive impairment compared with Asians. NHOPI had lower rate of advance directive, early DNR order, and any DNR order compared with Asians. Sub-analyses comparing the hematoma size for each ICH location showed that hematoma size was larger among Asians than NHOPI for lobar hemorrhage (lobar: Asians: 41 [15, 90] cm^3^ vs. NHOPI: 15 [4, 51] cm^3^, *p* = 0.03). However, no differences in the hematoma size was observed in the other locations (deep: Asians: 14 [5, 36] cm^3^ vs. NHOPI: 14 [7, 32] cm^3^, *p* = 0.85; cerebellum: Asians: 11 [6, 19] cm^3^ vs. NHOPI: 24 [10, 39] cm^3^, *p* = 0.31; brainstem: Asians: 5 [2, 11] cm^3^ vs. NHOPI: 3 [2, 8] cm^3^, *p* = 0.84).

**Table 1:** Comparison of characteristics between Asians and NHOPI.

	Asians	NHOPI	*p*
No. (%)	161	59	
Age, years	65 [51, 77]	52 [41, 65]	<0.0001
Female	68 (42.2)	24 (40.7)	0.84
Married	87 (54.0)	28 (47.5)	0.39
No health insurance	18 (11.2)	10 (17.2)	0.24
Annual household income <$15,000	38 (23.6)	22 (37.9)	0.04
Regular visit to a primary care physician	103 (67.3)	29 (50.0)	0.02
Hypertension	120 (74.5)	48 (81.4)	0.29
Diabetes mellitus	34 (21.1)	23 (39.0)	0.007
Hypercholesterolemia	64 (39.8)	22 (37.3)	0.74
Coronary artery disease	11 (6.8)	4 (6.8)	0.99
Atrial fibrillation	25 (15.5)	7 (11.9)	0.50
Obesity (BMI ≥ 30 kg/m^2^)	37 (23.1)	33 (58.9)	<0.0001
Coagulopathy (INR ≥ 1.5)	21 (13.0)	9 (15.3)	0.67
Dementia/cognitive impairment	15 (9.3)	0 (0)	0.02
Methamphetamine abuse	19 (11.8)	11 (18.6)	0.19
Initial systolic blood pressure, mmHg	172 [149, 198]	184 [157, 213]	0.12
Initial diastolic blood pressure, mmHg	96 [84, 111]	100 [86, 126]	0.11
Initial GCS, median [IQR]	13 [6, 15]	13 [6, 15]	0.95
Hematoma location			0.25
Deep (basal ganglia or thalamus)	87 (54.0)	28 (47.5)	
Lobar	48 (29.8)	17 (28.8)	
Cerebellum	16 (9.9)	5 (8.5)	
Brainstem	6 (3.7)	7 (11.9)	
Primary IVH	4 (2.5)	2 (3.4)	
IVH	74 (46.0)	23 (39.0)	0.36
Hematoma volume (cm^3^)	16 [7, 48]	14 [5, 37]	0.17
Advance directive present	47 (29.2)	4 (6.8)	0.0005
DNR order, early (<24 h)	39 (24.2)	2 (3.4)	0.0004
DNR order, any time during hospitalization	69 (42.9)	16 (27.1)	0.03
Withdrawal of life support	47 (29.2)	11 (18.6)	0.12
In-hospital mortality	53 (32.9)	12 (20.3)	0.07

*NHOPI, Native Hawaiians and Other Pacific Islanders; BMI, body mass index; INR, international normalized ratio; GCS, Glasgow Coma Scale; DNR, do-not-resuscitate; IVH, intraventricular hemorrhage*.

*Asians and NHOPI were compared with whites (reference group)*.

*Data are *n* (%), mean or median [IQR]*.

Overall, 65 (29.5%) achieved favorable outcome at 3 months. The comparison of mRS distribution between Asians and NHOPI is shown in Table 2 and demonstrates a significance difference between the two races. The prevalence of favorable 3-month outcome was higher among NHOPI compared with Asians (44.1 vs. 24.2%, respectively, *p* = 0.02) despite having similar ICH Score distribution. After excluding the in-hospital non-survivors, the difference in functional outcome between NHOPI and Asians persisted among the survivors of the initial hospitalization (55.3 vs. 36.1%, respectively, *p* = 0.03). Table 3 demonstrates the univariate and multivariable models. Compared with Asian race, NHOPI race was a predictor of favorable outcome in the unadjusted model (OR 2.47, 95% CI: 1.32–4.62) and after adjusting for the ICH Score (OR 2.30, 95% CI: 1.06–4.97) but not in the final model, which included ICH Score, early DNR order status and history of dementia/cognitive impairment (OR 2.04, 95% CI: 0.94–4.42). In this model, the ICH Score remained as the only independent negative predictor of outcome (OR 0.26, 95% CI: 0.17–0.41 per point).

**Table 2:** Comparison of ICH severity and outcome between Asians and NHOPI.

	Asians	NHOPI	*p*
ICH Score			0.40
0	35 (21.9)	19 (32.2)	
1	42 (26.3)	17 (28.8)	
2	38 (23.8)	10 (16.9)	
3	25 (15.6)	8 (13.6)	
4	15 (9.4)	2 (3.4)	
5	4 (2.5)	3 (5.1)	
6	1 (0.6)	0 (0)	

**Total cohort (*N* = 220)**			
mRS Score at 3 months			0.02
0	4 (2.5)	5 (8.5)	
1	26 (16.1)	14 (23.7)	
2	9 (5.6)	7 (11.9)	
3	23 (14.3)	6 (10.2)	
4	23 (14.3)	5 (8.5)	
5	11 (6.8)	8 (13.6)	
6	65 (40.4)	14 (23.7)	
Favorable outcome at 3 months (mRS ≤ 2)	39 (24.2)	26 (44.1)	0.004

**In-hospital survivors (*N* = 155)**			
mRS Score at 3 months			0.10
0	4 (3.7)	5 (10.6)	
1	26 (24.1)	14 (29.8)	
2	9 (8.3)	7 (14.9)	
3	23 (21.3)	6 (12.8)	
4	23 (21.3)	5 (10.6)	
5	11 (10.2)	8 (17.0)	
6	12 (11.1)	2 (4.3)	
Favorable outcome at 3 months (mRS ≤ 2)	39 (36.1)	26 (55.3)	0.03

*ICH, intracerebral hemorrhage; NHOPI, Native Hawaiians and Other Pacific Islanders; mRS, modified Rankin Scale*.

*Data are *n* (%)*.

**Table 3:** Multivariable model to predict favorable 3-month outcome (mRS ≤ 2) after ICH.

	Model 1OR (95% CI)	Model 2OR (95% CI)	Model 3OR (95% CI)
NHOPI race	2.47 (1.32, 4.62)^a^	2.30 (1.06, 4.97)^a^	2.04 (0.94, 4.42)
ICH Score (per 1 pt)		0.25 (0.16, 0.38)^a^	0.26 (0.17, 0.41)^a^
Early DNR order			0.28 (0.05, 1.50)
Dementia/cognitive impairment			0.46 (0.04, 4.90)

*mRS, modified Rankin Scale; ICH, intracerebral hemorrhage; OR, odds ratio; CI, confidence interval; NHOPI, Native Hawaiians and Other Pacific Islanders; DNR, do-not-resuscitate*.

*^a^Statistical significance*.

## Discussion

Our results showed that 29.5% of the ICH patients achieved favorable functional outcome at 3 months, which is consistent with the prior studies (1–3). Contrary to our hypothesis, NHOPI in our study were more likely to achieve favorable 3-month functional outcome compared with Asians after adjusting for the ICH Score. However, our observed impact of race on functional outcome after ICH was attenuated after adjusting for early DNR order and dementia/cognitive impairment, suggesting that the higher prevalence of these factors among Asians may have driven the observed racial differences in outcome. In the final model, the ICH Score remained as the only independent predictor of 3-month functional outcome as previously described (2). Despite a higher prevalence of cardiovascular risk factors and low socioeconomic status as shown in this study, functional outcome remained significantly better in NHOPI compared with Asians.

Our study emphasizes the importance of disaggregating racial and ethnic populations who may be historically grouped into a single racial/ethnic group but upon a more detailed analysis, suggests that other social or cultural factors (i.e., aggressiveness in clinical care) may underlie some of the observed differences in racial/ethnic outcomes (19, 20). Moreover, other unmeasured factors such as social support, spiritual, or cultural factors may have also influenced the differences in outcome and may be partly reflected in the aggressiveness of treatment after ICH.

Much of the differences in functional outcome could be explained by the racial differences in the aggressiveness of care that the patient’s families seek in the inpatient and post-discharge settings, as supported by the lower rate of early DNR, any DNR and advance directive presence among NHOPI compared with Asians. Also, there was a trend toward lower rate of withdrawal of life support among NHOPI compared with Asians. The lower rates of these measures often reflect the patients and families’ wishes to provide more aggressive and less palliative-oriented patient care. These decisions may be impacted by social, religious or cultural factors that may differ by race. It is possible that this outlook impacted the intensity and frequency of post-discharge rehabilitation processes for the NHOPI.

Although less aggressive care and higher prevalence of dementia/cognitive among the Asians may partially explain the observed racial differences in outcome, there may be other possible explanations. First, although we limited our studies to patients with baseline mRS of 0 (no symptoms), it is possible that older Asian group had lower baseline function than NHOPI, but were not captured by the prehospital mRS survey in this study. Second, there may be racial differences in the perception of disability after stroke and one group may score themselves higher on the disability rating despite having similar physical disabilities. Also, collective culture such as Asian culture strives for common social identify and may lead individuals with disabilities to perceive themselves as having greater burden to the society compared with those in more individualistic culture. Third, there may be fundamental biological differences between the two groups that impact functional outcome. For example, apolipoprotein E (APOE) 4 allele carriers have been associated with worse functional outcome after ICH compared with the non-carriers (21, 22), and therefore it is possible to have racial differences in the prevalence of APOE four allele carriers between Asians and NHOPI.

The strength of our study is that this is the largest prospective cohort study to characterize functional outcome after ICH among a diverse group of Asians and NHOPI. Prior ICH studies that assessed Asians and NHOPI in Hawaii were retrospective single-center studies that only assessed in-hospital mortality as an outcome measure (7). This study recruited a relatively large number of NHOPI population, which has been historically underrepresented in prior prospective stroke studies. The limitation of this study is the relatively low sample size, which led to the elimination of whites from the analyses. Also, since this is a single-center study, generalizability of our results to other populations may be limited. Specifically, because our institution is a tertiary referral center, there may have been a referral bias toward more severe ICH patients since those with small hematomas and minor neurological symptoms may not have been transferred to our facility. Also, it is possible that some of the older ICH patients with preexisting DNR orders or those with terminal illness may not have transferred to our facility, creating a possible selection bias toward younger ICH patients. We also did not completely account for the age factor in the model since dichotomized age factor (<80 vs. ≥80 years) used in the ICH Score may not necessarily distinguish the disparate number of very young population. Finally, we cannot exclude the possibility that this cohort study enrolled ICH patients with different severity mix compared with other published studies. However, 65 of 220 patients (29.5%) achieved mRS score ≤2 at 3 months, which is comparable to other observational studies (1–3). This study showed that after ICH, NHOPI are more likely to achieve favorable functional outcome compared with Asians even after adjusting for the severity of ICH. However, this difference was attenuated after adjusting for the DNR and baseline cognitive status. This study further supports efforts to disaggregate Asians and NHOPI from the traditionally combined “Asian American Pacific Islander” classification, which may mask potentially different underlying mechanisms as well as cultural differences between Asians and NHOPI in patient and family responses to severely disabling conditions such as ICH.

## Ethics Statement

This study was approved by the University of Hawaii Institutional Review Board, and written informed consent was obtained from the patient or legally authorized surrogate decision-maker.

## Author Contributions

KN participated in the conception and design of the study, the acquisition of data, the analysis and interpretation of data, and was responsible for drafting and finalizing the manuscript. SK participated in the acquisition of data and helped to draft and finalize the manuscript. TS participated in the study supervision, analysis and interpretation of data, and was responsible for drafting and finalizing the manuscript. MM participated in the study supervision, analysis and interpretation of data, and was responsible for drafting and finalizing the manuscript.

## Conflict of Interest Statement

The authors declare that the research was conducted in the absence of any commercial or financial relationships that could be construed as a potential conflict of interest.
